# Generation of correlated biphoton via four-wave mixing coexisting with multi-order fluorescence processes

**DOI:** 10.1038/s41598-019-56567-9

**Published:** 2019-12-27

**Authors:** Yang Liu, Kangkang Li, Siqiang Zhang, Huanrong Fan, Wei Li, Yanpeng Zhang

**Affiliations:** 1 Key Laboratory for Physical Electronics and Devices of the Ministry of Education & Shaanxi Key Laboratory of Information Photonic Technique, Xi’an Jiaotong University, Xi’an, 710049 China; 2 State Key Laboratory of Transient Optics and Photonics, Xi’an Institute of Optics and Precision Mechanics, Chinese Academy of Sciences, Xi’an, 710119 China; 3 University of Chinese Academy of Sciences, Beijing, 100049 China

**Keywords:** Atom optics, Nonlinear optics

## Abstract

We investigate the parametrically amplified four-wave mixing, spontaneous parametric four-wave-mixing, second- and fourth-order fluorescence signals coming from the four-level double-Λ electromagnetically induced transparency system of a hot ^85^Rb atomic vapor. The biphoton temporal correlation is obtained from spontaneous parametric four-wave-mixing and fourth-order fluorescence processes. Meanwhile, we first observed the biphoton Rabi oscillation with a background of linear Rayleigh scattering and uncorrelated second-order fluorescence. The outcomes of the investigation may contribute potentially to the applications in dense coding quantum communication systems.

## Introduction

In recent five decades, the generation of time-energy entangled photon pairs has attracted worldwide attention, because these correlations are central to the foundational questions in quantum mechanics^1^ and play a vital role in application oriented research of quantum communication^2^, computation^3^, quantum imaging^4,5^, and quantum metrology^6,7^. Generally, the correlated photon pairs are generated by spontaneous parametric down-conversion (SPDC) in nonlinear crystals^8,9^. However, the photon pairs from this nonlinear process usually have wide bandwidth (THz), short coherence time (ps) and short coherence length (100 *μ*m), which comes as a limitation for long-distance fiber optical quantum communication^10^. To solve this problem, Du’s group generated subnatural-linewidth correlated biphoton from the spontaneous parametric four-wave mixing (SP-FWM) in the cold atoms (10–100 *μ*K)^11–14^. SP-FWM nonlinear process can produce narrow-band (MHz) and ultra-long coherence time (*μ*s) two-mode entanglement source. Moreover, an SP-FWM process can generate correlated photon pairs of Stokes (*E*_*S*_) and anti-Stokes (*E*_*aS*_) coexisting with multi-order fluorescence (*FL*) and Rayleigh scattering signals simultaneously. In addition, *E*_*S*_ and *E*_*aS*_ can also be used in an optical parametric amplification (OPA) process to research the squeezed and entangled states of optical fields^15–20^. Currently, a great deal of work has been done in studying the mechanism of the nonlinear optical process, such as the influence of dressing fields on parametric amplification of four-wave mixing (PA-FWM) processes in a “double-Λ” atomic system^21^.

In this paper, we propose the experimental demonstration of the generation of narrow-bandwidth nondegenerate paired photons from a hot ^85^Rb atomic ensemble via coexisting SP-FWM and multi-fluorescence. We observed the biphoton Rabi oscillations with a background of linear Rayleigh scattering and second-order fluorescence. These nonlinear optical processes are controlled by adjustable detuning of pump and coupling fields. The outcomes of the investigation may potentially contribute to the applications in dense coding quantum communication systems. The paper is constructed as follows: in section II, firstly we study the generation processes of PA-FWM, SP-FWM and multi-order fluorescence. Then calculate the coincidence counting of the SP-FWM and correlation of multi-order fluorescence. In section III, we study the influence between the detuning of pump field and EIT windows. Then discuss the biphoton correlations of SP-FWM and multi-order fluorescence. In section IV, we conclude the paper.

## Basic Theory

**Figure 1:**
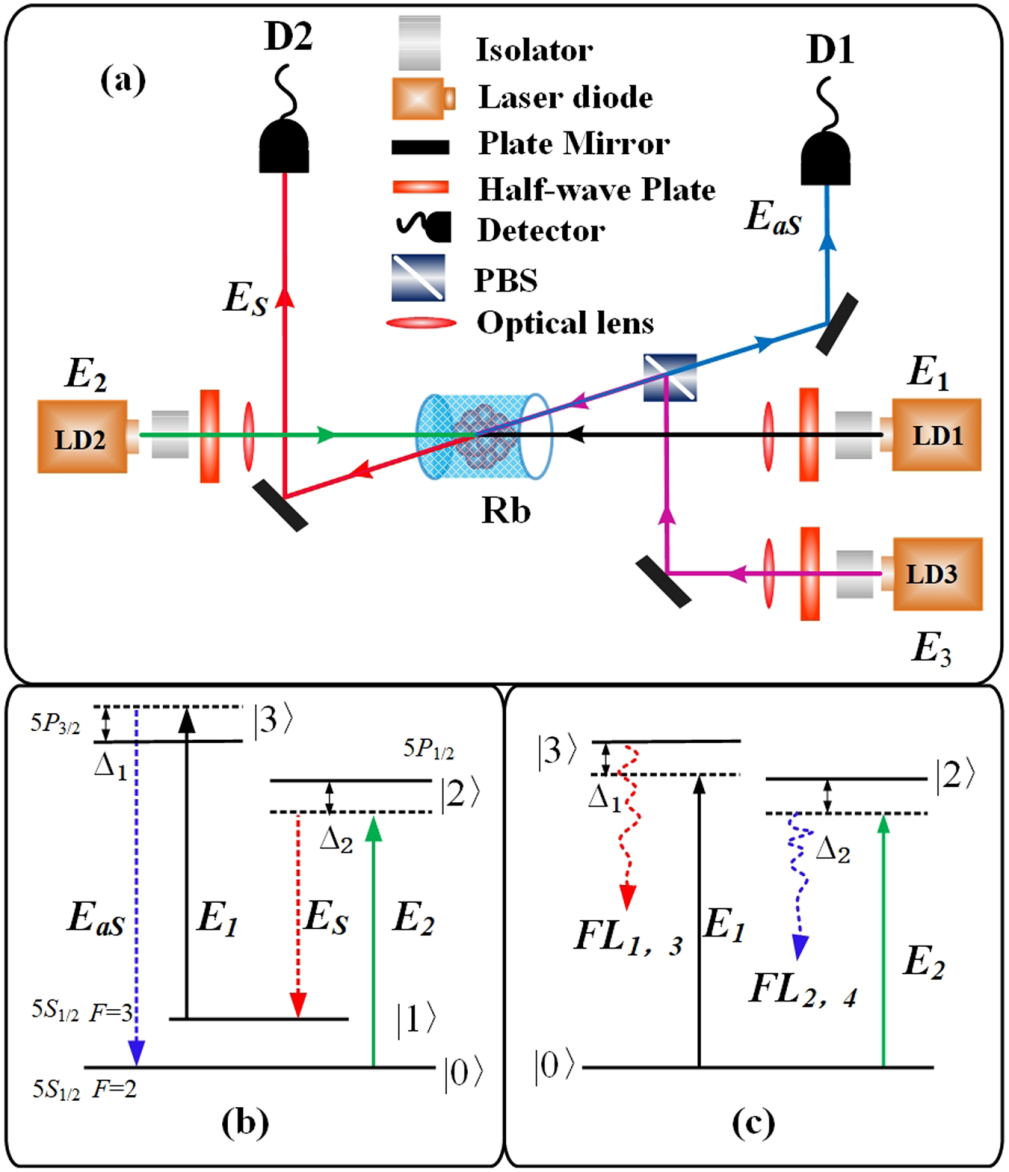
(**a**) The experimental setup and spatial beams alignment of the FWM and fluorescence processes; I: isolator; LD1-3: laser diode; PBS: polarization beam splitter; D1-2: avalanche photodiode detector (APD) or single-photon counting module (SPCM). (**b**) The energy diagrams of FWM generation processes in “double-Λ” four-level atomic systems of ^85^Rb, respectively. *E*_*s*_ and *E*_*aS*_ denote the Stokes and anti-Stokes signals. (**c**) The energy diagrams of fluorescence generation processes. *FL*_1,2_ and *FL*_3,4_ denote the second- and fourth-order fluorescence signals.

We start the experiment description with the spatial beams alignment and associated energy level diagram of the FWM and fluorescence processes shown in Fig. 1. The experiments are carried out in a “double-Λ” four-level atomic systems of ^85^Rb shown in Fig. 1(b). The |0〉 (5*S*_1/2_, F = 2), |1〉 (5*S*_1/2_, F = 3), |2〉 (5*P*_1/2_) and |3〉 (5*P*_3/2_) are four relevant atomic energy levels. The strong pump laser beam *E*_1_ (frequency *ω*_1_, wave vector **k**_1_, Rabi frequency *G*_1_, wavelength 780 nm, power up to 54.5 mW) connecting |0〉 (5*S*_1/2_, F = 3) and |3〉 (5*P*_3/2_) comes from the laser diode 1 (LD1). The coupling laser beam *E*_2_ (*ω*_2_, **k**_2_, *G*_2_, 795 nm, 39 mW) connecting |1〉(5*S*_1/2_, F = 2) and |2〉 (5*P*_1/2_) is emitted by the LD2. The weak probe laser beam *E*_3_ (*ω*_3_, **k**_3_, *G*_3_, 780 nm, 7.2 mW) connecting |3〉 (5*P*_3/2_) and |1〉 (5*S*_1/2_, F = 3) comes from the LD3 in the *E*_1_ direction. As indicated in Fig. 1(a), the incident beam *E*_1_ propagates in the same direction with *E*_3_, and can form a standard Λ-EIT window, while the *E*_1_ propagates in the opposite direction with *E*_2_, which also generate a new type EIT window. The FWM signals and transmitted probe beam in Fig. 1(c) are detected by an avalanche photodiode detector (APD) and satisfy the phase matching condition (PMC) of **k**_*aS*_ = **k**_1_ + **k**_2_ − **k**_*S*_. In addition, if we block the injection laser *E*_3_ and change the detectors for two single-photon counting modules (SPCM), the biphoton coincidence counts with fluorescence signals can be detected.

## Generation Process of SP-FWM and Coincidence Counting

Firstly, we block the injection laser *E*_3_ to get the SP-FWM (*E*_*S*_ and *E*_*aS*_) which can be described by the third-order density matrix elements (the solutions are given in the Methods). The phase matching condition (${{\bf{k}}}_{1}+{{\bf{k}}}_{2}={{\bf{k}}}_{S}+{{\bf{k}}}_{aS}$) of the two spontaneous emission signals is satisfied. Generally, we assume that the *E*_*S*_ and *E*_*aS*_ fields are much weaker than the *E*_1_ and *E*_2_, so they are regarded as two classical fields (*E*_1_ and *E*_2_) and two quantum fields (described as *a*^†^ and *b*^†^) with the Hamiltonian $H=i\hslash \kappa {\hat{a}}^{\dagger }{\hat{b}}^{\dagger }+h.\,{\rm{c}}.$, respectively. Where *κ* is the nonlinear coefficient and can be expressed as $\kappa =|{\chi }_{S/aS}^{(3)}{E}_{1}{E}_{2}|=|N{\mu }_{10}^{2}{\rho }_{S/aS}^{(3)}/\hslash {\varepsilon }_{0}{G}_{S/aS}|$. According to the perturbation theory, it can be described as the models of the *E*_*S*_ and *E*_*aS*_, the correspondings perturbation chains are ${\rho }_{11}^{(0)}\mathop{\to }\limits^{{\omega }_{1}}{\rho }_{31}^{(1)}\mathop{\to }\limits^{-{\omega }_{aS}}{\rho }_{01}^{(2)}\mathop{\to }\limits^{{\omega }_{2}}{\rho }_{21(S)}^{(3)}$ and ${\rho }_{00}^{(0)}\mathop{\to }\limits^{{\omega }_{2}}{\rho }_{20}^{(1)}\mathop{\to }\limits^{-{\omega }_{S}}{\rho }_{10}^{(2)}\mathop{\to }\limits^{{\omega }_{1}}{\rho }_{30(aS)}^{(3)}$ respectively.

The propagation equation of Stokes and anti-Stokes photons can be written as: $\frac{d}{dt}\hat{a}=k{\hat{b}}^{\dagger }$ and $\frac{d}{dt}\hat{b}=k{\hat{a}}^{\dagger }$. where *a*^†^ and *b*^†^ are creation operators for Stokes and anti-Stokes photons, respectively. Solving these above two equations, we can obtain the output Stokes and anti-Stokes fields as: $\hat{a}(t)=\,\cosh (kt)\hat{a}(0)+\,\sinh (kt){\hat{b}}^{\dagger }(0)$ and ${\hat{b}}^{\dagger }(t)=\,\sinh (kt)\hat{a}(0)+\,\cosh (kt){\hat{b}}^{\dagger }(0)$. The interaction of Hamiltonian determines the evolution of the two-photon state vector^22^. The mechanism of biphotons generation near the resonance is explained clearly^23^. The biphoton amplitude in the time domain can be expressed as:

1 $$\psi (\tau )=\frac{L}{2\pi }\int d{\omega }_{as}\kappa ({\omega }_{as})\Phi ({\omega }_{as}){e}^{-i{\omega }_{as}\tau }.$$

where Φ(*ω*_*as*_) is the longitudinal detuning function, and can be written as $\Phi ({\omega }_{as})={\rm{sinc}}(\frac{\Delta kL}{2}){e}^{i\frac{L}{2}[{k}_{s}({\omega }_{as})+{k}_{as}({\omega }_{as})]}$, and the relative time delay $\tau ={t}_{aS}-{t}_{S}$. The biphotons wave function is determined by both the longitudinal detuning function and nonlinear coupling coefficient. The third-order nonlinear susceptibility of the anti-Stokes field can be defined as:

2 $${\chi }_{aS}^{(3)}=\frac{N{\mu }_{13}{\mu }_{14}{\mu }_{23}{\mu }_{24}}{{\varepsilon }_{0}{\hslash }^{3}}\frac{1}{({\Gamma }_{20}+{{\rm{i}}\Delta }_{2})({\Gamma }_{10}+{\rm{i}}\delta )({\Gamma }_{30}+{\rm{i}}\delta +{{\rm{i}}\Delta }_{1})}.$$

where *μ*_*ij*_ are the electric dipole matrix elements and Γ_*ij*_ are the dephasing rates of coherence $|i\rangle \to |j\rangle$. ${\Delta }_{1}={\omega }_{31}-{\omega }_{1}$ and ${\Delta }_{2}={\omega }_{20}-{\omega }_{2}$ are the detunings of coupling and pump field. The damped oscillation with a frequency of Δ_1_ results from the interference between two resonance bands at *δ* = 0 and Δ_1_. The two-photon coincidence counting rate can be calculated as:

3 $$Rcc(\tau )=W[1-\,\cos ({\Delta }_{1}\tau )]{e}^{-2{\Gamma }_{e}\tau }.$$

where *W* is a constant, ${\tau }_{r}=2\pi /{\Delta }_{1}$ is the Rabi period, ${\Gamma }_{e}=({\Gamma }_{10}+{\Gamma }_{30})/2$ is the effective dephasing rate and ${\tau }_{e}={\Gamma }_{e}/2$ is the nonlinear coherence time.

## Generation and Correlation of Multi-Order Fluorescence

In the system, the second-order fluorescence is generated through the perturbation chains ${\rho }_{00}^{(0)}\,\mathop{\to }\limits^{{G}_{1}}\,{\rho }_{30}^{(1)}\,\mathop{\to }\limits^{{G}_{1}^{\ast }}\,{\rho }_{33(FL1)}^{(2)}$ and ${\rho }_{00}^{(0)}\,\mathop{\to }\limits^{{G}_{2}}\,{\rho }_{20}^{(1)}\,\mathop{\to }\limits^{{G}_{2}^{\ast }}\,{\rho }_{22(FL2)}^{(2)}$ shown in Fig. 1(c). The diagonal density matrix elements are ${\rho }_{33(FL1)}^{(2)}=\frac{-{G}_{1}^{2}}{{(\Gamma }_{30}+i{\Delta }_{1}{)\Gamma }_{33}}$ and ${\rho }_{22(FL2)}^{(2)}=\frac{-{G}_{2}^{2}}{{(\Gamma }_{20}+i{\Delta }_{2}{)\Gamma }_{22}}$. When *E*_1_ and *E*_1_ are on, the second-order fluorescence with dressing fields *E*_1_ and *E*_1_ are rewritten as follows:

4 $${\rho }_{33D}^{(2)}=\frac{-{G}_{1}^{2}}{({\Gamma }_{30}+i{\Delta }_{1}+\frac{{G}_{2}^{2}}{{\Gamma }_{32}+i({\Delta }_{1}-{\Delta }_{2})}){\Gamma }_{33}},\,{\rho }_{22D}^{(2)}=\frac{-{G}_{2}^{2}}{({\Gamma }_{20}+i{\Delta }_{2}+\frac{{G}_{1}^{2}}{{\Gamma }_{23}+i({\Delta }_{2}-{\Delta }_{1})}){\Gamma }_{22}}.$$

Then, the fourth-order fluorescence is generated through the perturbation chains ${\rho }_{00}^{(0)}\,\mathop{\to }\limits^{{G}_{2}}\,{\rho }_{20}^{(1)}\,\mathop{\to }\limits^{{G}_{2}^{\ast }}\,{\rho }_{00}^{(2)}\,\mathop{\to }\limits^{{G}_{1}}\,{\rho }_{30}^{(3)}\,\mathop{\to }\limits^{{G}_{1}^{\ast }}\,{\rho }_{33(FL3)}^{(4)}$ and ${\rho }_{00}^{(0)}\,\mathop{\to }\limits^{{G}_{1}}\,{\rho }_{30}^{(1)}\,\mathop{\to }\limits^{{G}_{1}^{\ast }}\,{\rho }_{00}^{(2)}\,\mathop{\to }\limits^{{G}_{2}}\,{\rho }_{20}^{(3)}\,\mathop{\to }\limits^{{G}_{2}^{\ast }}\,{\rho }_{22(FL4)}^{(4)}$.

The diagonal density matrix element and given as follows:

5 $${\rho }_{33(FL3)}^{(4)}=\frac{{G}_{1}^{2}{G}_{2}^{2}}{{(\Gamma }_{20}+i{\Delta }_{2}{)\Gamma }_{00}{(\Gamma }_{30}+i{\Delta }_{1}{)\Gamma }_{33}},\,{\rho }_{22(FL4)}^{(4)}=\frac{{G}_{2}^{2}{G}_{1}^{2}}{{(\Gamma }_{30}+i{\Delta }_{1}{)\Gamma }_{00}{(\Gamma }_{20}+i{\Delta }_{2}{)\Gamma }_{22}}$$

The fluorescence propagation equation is *I* = *I*_*FL*_ − *I*_*A*_, where ${I}_{FL}=C{N}_{FL}^{2}\,{\mu }^{2}\,{\int }_{-\infty }^{+\infty }\,({e}^{-{(v/u)}^{2}}|{\rho }^{(4)}(v){|}^{2}\,/\,u\sqrt{\pi })dv$ is the total intensity of the generated fluorescence signal. ${\rho }^{(4)}(\nu )$ is the density-matrix element of the fluorescence signal including pure fluorescence and multi-order fluorescence signals. *I*_*A*_ is the absorption of the fluorescence signals in the medium and may be written as ${I}_{A}={I}_{FL}(1-{e}^{-\alpha L})=C{N}_{FL}^{2}\,{\mu }^{2}\,{\int }_{-\infty }^{+\infty }\,({e}^{-{(v/u)}^{2}}K\,{\rm{Im}}[FL(v)]/u\sqrt{\pi })dv$. Where *α* is the absorption co-efficient. *C* is a constant. $K={I}_{0}L{k}_{1}/C{N}_{FL}\hslash {\varepsilon }_{0}$. $F=\hslash {\varepsilon }_{0}\chi /{N}_{FL}{\mu }^{2}$ is the effective atom number. *μ* is the dipole moment. *v* is the velocity of the atom due to Doppler effect. *u* is the most probable velocity. For the coupling field fluorescence, the intensities of fluorescence are proportional to the ${\rho }_{33(FL)}^{(4)}$ and ${\rho }_{22(FL)}^{(4)}$, where the brackets express the time average $\langle {I}_{i}(t)\rangle ={\int }_{t}^{t+T}\,{I}_{i}(t)/T$, 〈*I*_*i*_〉 is the average intensity of each laser beam and *I*_*i*_(*t*) gives the intensity versus time. *T* is the time of integration. ${I}_{i}(t)\approx {\Omega }_{i}^{2}-2{\Omega }_{i}{\eta }_{i}L\,{\rm{Im}}[{\rho }_{31i}^{(1)}(t)]$ and ${I}_{j}(t)\approx {\Omega }_{j}^{2}-2{\Omega }_{j}{\eta }_{j}L\,{\rm{Im}}[{\rho }_{32j}^{(1)}(t)]\,$are the intensities in this process. The correlations between the fluorescence *I*_*m*_ and *I*_*n*_ are given by the *G*^(2)^(*τ*), which is a function between time delay *τ* and the intensities^24^:

6 $$\begin{array}{rcl}{G}^{(2)}(\tau ) & = & \frac{\langle {I}_{m}(t){I}_{n}(t+\tau )\rangle }{\sqrt{\langle {[{I}_{m}(t)]}^{2}\rangle \langle {[{I}_{n}(t+\tau )]}^{2}\rangle }}\\  & = & \frac{\langle \{{\Omega }_{m}^{2}-2{\Omega }_{m}{\eta }_{m}L\,{\rm{Im}}[{\rho }_{m}^{(1)}(t)]\}\{{\Omega }_{n}^{2}-2{\Omega }_{n}{\eta }_{n}L\,{\rm{Im}}[{\rho }_{n}^{(1)}(t+\tau )]\}\rangle }{\sqrt{\langle {\{{\rm{Im}}[{\rho }_{m}^{(1)}(t)]\}}^{2}\rangle \langle {\{{\rm{Im}}[{\rho }_{n}^{(1)}(t+\tau )]\}}^{2}\rangle }}\\  & \propto  & 1+\,{{\rm{sinc}}}^{2}(\frac{\Delta \omega \tau }{2\pi })\end{array}$$

## Results and Discussion

**Figure 2:**
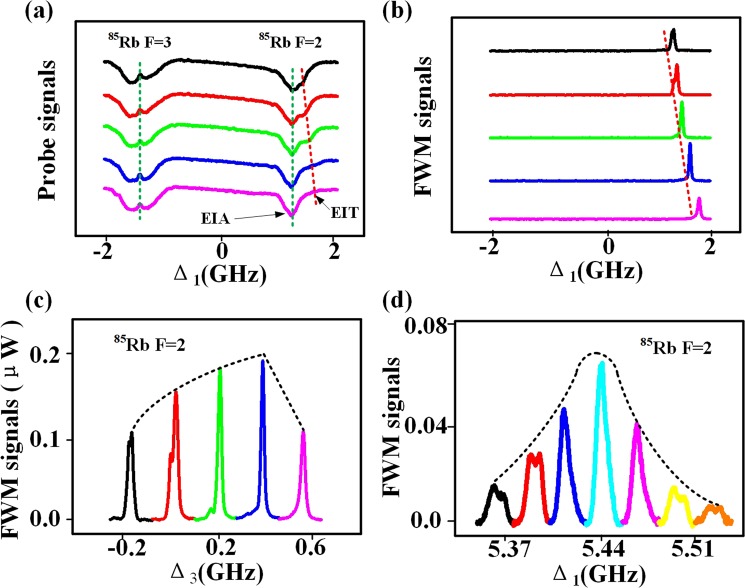
(**a**) By fixing the wavelength of *E*_2_ at 794.981 nm, the probe transmission spectra versus Δ_1_ which the five curves from top to bottom are obtained with increased Δ_3_. The red and green dotted lines are the fit lines of the EIT and EIA. (**b**) Measured FWM signals versus Δ_1_ at discrete Δ_3_ correspondings to (**a**). (**c**) The FWM signals *E*_*F*1_ with increased Δ_3_ from left to right corresponding to (**b**). The black dotted line is the fit line of the FWM signals. (**d**) The FWM signals *E*_*F*1_ with increased Δ_1_.

First, the wavelength of the strong coupling laser *E*_2_ was fixed at 794.981 nm, which connects the |1〉 (5*S*_1/2_, F = 3) and |2〉 (5*P*_1/2_) transition of the ^85^Rb D1 line in Fig. 1(a). The frequency of the pump laser *E*_1_ was monitored and scanned over the entire range of ground and excited states. By changing the detuning Δ_3_ from −0.2 to 0.6 GHz, we observed the positions of standard Λ-EIT window (*E*_1_ and *E*_3_ satisfying Δ_1_ − Δ_3_ = 0) on the typical probe transmission spectrum in Fig. 2(a) and the intensity variation of FWM signals detected by the APD1 in Fig. 2(b). While the Λ-EIT windows are moved in the positive direction along Δ_1_-axis by the increasing Δ_3_. Five sharp peaks of *E*_*F*1_ on the FWM spectrum are observed falling into the Λ-EIT windows corresponding to Fig. 2(a). The phenomenon indicates that the primary cause of *E*_*F*1_ switches is atomic coherence. Additionally, we use the saturated absorption technique and EIT peaks to calibrate the positions of the coupling and pump beams on the probe transmission spectrum. The windows can be identified by fixing the different incident beams and increasing detuning of the other laser beams. The intensity of the FWM signals can be controlled easily by adjusting the detuning of the *E*_3_ in Fig. 2(c). The maximum enhancement of the *E*_*F*1_ signal is approximately 0.2 *μ*W when Δ_3_ = 0.4 GHz is satisfied.

**Figure 3:**
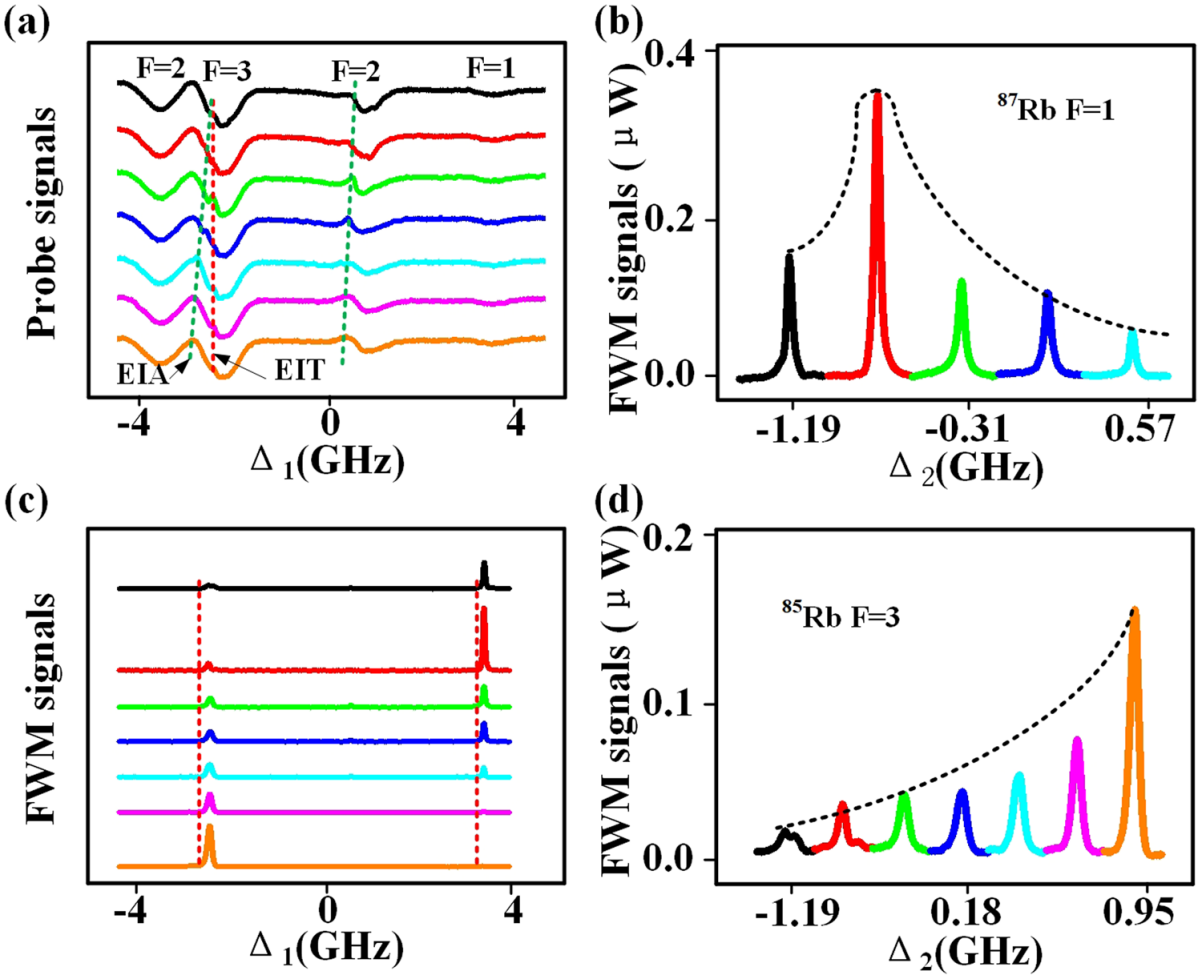
(**a**) By fixing the wavelength of *E*_3_ at 780.237 nm, the probe transmission spectrum versus Δ_1_ which the seven curves from top to bottom are obtained with increasing Δ_2_. The red and green dotted lines are the fit lines of the EIT and EIA. (**b**) The FWM signals *E*_*F*2_ of ^87^Rb F = 1 with increased Δ_2_ from left to right. The black dotted line is the fit line of the FWM signals. (**c**) Measured FWM signals versus Δ_1_ at discrete Δ_2_ corresponding to (**a**). (**d**) The FWM signals *E*_*F*3_ of ^85^Rb F = 3 with increased Δ_2_ from left to right.

In the following, we show the effect of each window by scanning the detuning Δ_1_ at different detuning Δ_2_. This method is very convenient for the observation of suppression and enhancement. The measured probe curves versus Δ_1_ shown in Fig. 3(a), where the seven curves from top to bottom are obtained with increased Δ_2_. Corresponding to Fig. 2(a), the measured FWM curves versus Δ_1_ at discrete Δ_2_ are shown in Fig. 3(c). We observed the FWM signal *E*_*F*2_ and *E*_*F*3_ from the “double Λ” four-level atomic systems with fixed beam *E*_3_ (780.237 nm). The Λ-EIT windows formed by *E*_1_ and *E*_3_ in the dip of F = 3 does not move with the increase of Δ_2_ in Fig. 3(a). While the new type EIT windows are moved in the negative direction along Δ_1_-axis. Since, the detuning of probe beams is fixed, the peaks of *E*_*F*2_ and *E*_*F*3_ are also fixed in Fig. 3(c). Additionally, we use the saturated absorption technique and EIT peaks to calibrate the positions of the coupling and pump beams on the probe transmission spectrum. Similarly, the windows can also be identified by fixing the different incident beams and increasing detuning of the other laser. The intensity of the FWM signals can be controlled easily by adjusting the detuning of the coupling beam in Fig. 3(b,d). The maximum enhancement of the *E*_*F*3_ signal is approximately 0.35 *μ*W when Δ_2_ = −0.75 GHz is satisfied. When Δ_2_ changes from −1.19 to 0.95 GHz discretely, the *E*_*F*2_ signal increased to 0.15 *μ*W. Moreover, the linewidth of each window is narrow, the induced suppression (or enhancement) of *E*_*F*1_ is very sensitive to the relative position between each other.

At last, we block the injection laser *E*_3_ and fix the wavelength of *E*_1_ and *E*_2_ at 780.2396 and 794.9828 nm respectively. Then adjust the detectors into two SPCMs. The Stokes and anti-Stokes paired photons generated from the SP-FWM nonlinear process simultaneously, which propagate in opposite direction in Fig. 1(a) in the hot atomic ensemble. The Stokes photons usually propagate through the atomic transitions with the speed of light in vacuum *c*. And the anti-Stokes photons propagate through a coherent Λ-EIT window which determines the paired photons correlation time and waveforms. Simultaneously, the biphoton coincidence counts are detected by two independent SPCMs and recorded by a time-to-digit converter with a temporal bin width of 0.0244 ns. The measurement results of the signals ineluctably include the other three parts: the correlated fourth-order fluorescence, linear Rayleigh scattering and uncorrelated second-fluorescence.

We observed the FWM signal *E*_*F*2_ and *E*_*F*3_ from the “double Λ” four-level atomic systems with fixed beam *E*_3_ (780.237 nm). The Λ-EIT windows formed by *E*_1_ and *E*_3_ in the dip of F = 3 does not move by increasing the Δ_2_ in Fig. 3(a). While the new type EIT windows are moved in the negative direction along Δ_1_-axis. Since the detuning of probe beams is fixed, the peaks of *E*_*F*2_ and *E*_*F*3_ are also fixed in Fig. 3(c). Additionally, we use the saturated absorption technique and EIT peaks to calibrate the positions of the coupling and pump beams on the probe transmission spectrum. Similarly, the windows can also be identified by fixing the different incident beams and increasing detuning of the other laser. The intensity of the FWM signals can be controlled easily by adjusting the detuning of the coupling beam in Fig. 3. The maximum enhancement of the *E*_*F*3_ signal is approximately 0.35 *μ*W when Δ_2_ = −0.75 GHz is satisfied. When Δ_2_ changes from −1.19 to 0.95 GHz discretely, the *E*_*F*2_ signal monotonically increased to 0.15 *μ*W. Moreover, the linewidth of each window is narrow, the induced suppression (or enhancement) of *E*_*F*1_ is very sensitive to the relative position between each other.

**Figure 4:**
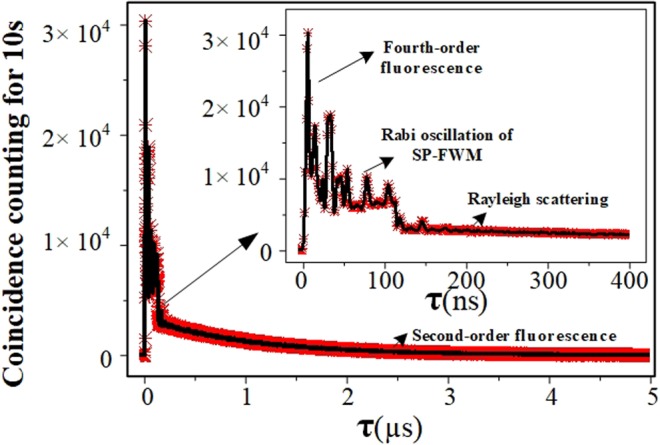
By fixing the wavelength of *E*_1_ and *E*_2_ at 780.2396 nm and 794.9828 nm respectively, biphoton coincidence counts as function of relative time delay *τ* between paired Stokes and anti-Stokes and multi-order fluorescence photons collected over 10 s with 0.0244 ns bin width.

Physically, this waveform can be explained as follows: we calculate the coherence time of the different paired photons to distinguish their contribution to the result of coincidence counts. The effective dephasing rate of *E*_*S*_ and *E*_*aS*_ is ${\Gamma }_{e}=({\Gamma }_{10}+{\Gamma }_{30})/2$, thus their coherence time is 116.3 ns shown in the Eq. 3, which agrees well with the experiment result in Fig. 4. While the coherence time of fourth-order fluorescence is 18.2 ns shown in the Eq. 6. So it is easy to distinguish these two paired photons. The background nonzero floor is a result of accidental coincidence between the on-resonance Rayleigh scattering, the second-order fluorescence and uncorrelated Stokes and anti-Stokes photons from different pairs. These photons have the same polarization and central frequency as the Stokes and anti-Stokes photons. So they cannot be filtered away by the polarization and frequency filters.

## Conclusion

In conclusion, we used hot atomic-gas media to generate non-classical light through the SP-FWM process in four-energy level system, especially focusing on biphoton generation. Our work shows that the pump and coupling field corporately determine the EIT. The EIT dephasing rate and loss determined the biphoton correlation time and waveforms. Meanwhile, we also observed the biphoton Rabi oscillation of SP-FWM with correlation time 116.3 ns. The background was attributed to linear Rayleigh scattering and uncorrelated second-fluorescence. We experimented with the method of using hot atoms to analyze and suppress the influence of the noise term, that is, the uncorrelated terms. This work pave the way for finding suitable modules for quantum communication.

## Methods

### Experimental setup

The experiments are carried out in a “double-Λ” four-level (|0〉 (5*S*_1/2_, F = 2), |1〉 (5*S*_1/2_, F = 3), |2〉 (5*P*_1/2_) and |3〉 (5*P*_3/2_)) atomic systems of ^85^Rb shown in Fig. 1. The strong pump laser beam *E*_1_, the coupling beam *E*_2_ and the weak probe laser beam *E*_3_ are emitted by the LD1-3 with the diameter 0.2 mm, respectively. The angle between the beams *E*_1_ and *E*_3_ is 0.26°. A thermal temperature-stabilized rubidium vapor cell with length of *L* = 5.5 cm is heated up 55 °C in center of this experiment setup and the atom density is about 2.5 × 10^11^ cm^−3^ in order to have enough atoms in the cavity to enhance the strength of atom-cavity coupling. Blocking the injection laser *E*_3_ and using two detectors (avalanche diode, PerkinElmer SPCM-AQR-15-FC, 50% efficiency, maximum dark count rate of 50/s)), the biphoton coincidence counts with fluorescence signals can be detected.

### Third-order density matrix elements of PA-FWM

When *E*_3_ is injected into the Stokes port of SP-FWM process in stokes channel, the generated *E*_*S*_ and *E*_*aS*_ signals in PA-FWM can be described by the third-order density matrix elements, which could be obtained by the perturbation chains ${\rho }_{11}^{(0)}\,\mathop{\to }\limits^{{\omega }_{1}}\,{\rho }_{31}^{(1)}\,\mathop{\to }\limits^{-{\omega }_{aS}}\,{\rho }_{01}^{(2)}\,\mathop{\to }\limits^{{\omega }_{2}}\,{\rho }_{21(S)}^{(3)}$ and ${\rho }_{00}^{(0)}\,\mathop{\to }\limits^{{\omega }_{2}}\,{\rho }_{20}^{(1)}\,\mathop{\to }\limits^{-{\omega }_{3}}\,{\rho }_{10}^{(2)}\,\mathop{\to }\limits^{{\omega }_{1}}\,{\rho }_{30(aS)}^{(3)}$:

7 $${\rho }_{21(S)}^{(3)}=-i{G}_{1}{G}_{aS}{G}_{2}/{d}_{11}{d}_{31}{d}_{01},\,{\rho }_{20(aS)}^{(3)}=-i{G}_{2}{G}_{3}{G}_{1}/{d}_{20}{d}_{10}{d}_{30}.$$

where ${G}_{i}={\mu }_{ij}{E}_{i}/\hslash (i,j=1,2,3,aS)$ is the Rabi frequency between levels $|i\rangle \leftrightarrow |j\rangle$, and *μ*_*ij*_ is the dipole momentum; ${d}_{30}={\Gamma }_{30}+i({\Delta }_{2}-{\Delta }_{3}+{\Delta }_{1})$, ${d}_{11}={\Gamma }_{11}+i{\Delta }_{1}$, ${d}_{31}={\Gamma }_{31}$, ${d}_{01}={\Gamma }_{01}+i{\Delta }_{2}$, ${d}_{20}={\Gamma }_{20}+i{\Delta }_{2}$, ${d}_{10}={\Gamma }_{10}+i({\Delta }_{2}-{\Delta }_{3})$, ${\Gamma }_{ij}=({\Gamma }_{i}+{\Gamma }_{j})/2$ is the decoherence rate between |*i*〉 and |*j*〉; ${\Delta }_{i}={\Omega }_{i}-{\omega }_{i}$ is detuning defined as the difference between the resonant transition frequency Ω_*i*_ and the laser frequency *ω*_*i*_ of *E*_*i*_.

The photon numbers of the output Stokes and anti-Stokes fields of optical parametric amplification can be described as:

8 $$\langle {N}_{a}\rangle =\langle { {\hat{a}} }_{out}^{\dagger }{ {\hat{a}} }_{out}\rangle =g\langle { {\hat{a}} }_{in}^{\dagger }{ {\hat{a}} }_{in}\rangle +(g-1),\,\langle {N}_{b}\rangle =\langle {\hat{b}}_{out}^{\dagger }{\hat{b}}_{out}\rangle =(g-1)\langle { {\hat{a}} }_{in}^{\dagger }{ {\hat{a}} }_{in}\rangle +g.$$

where $g=\{\cos \,[2t\sqrt{AB}\,\sin ({\varphi }_{1}+{\varphi }_{2})/2]+\,\cosh \,[2t\sqrt{AB}\,\cos ({\varphi }_{1}+{\varphi }_{2})/2]\}/2$ is the dressed SP-FWM gain with the modulus *A* and *B* (phase angles *φ*_1_ and *φ*_2_) defined in ${\rho }_{21(S)}^{(3)}=A{e}^{i{\varphi }_{1}}$ and ${\rho }_{20(aS)}^{(3)}=B{e}^{i{\varphi }_{2}}$, respectively.

## Data Availability

The data is available from the corresponding author on reasonable request.
